# Fever Nursing

**Published:** 1899-09

**Authors:** 


Fever Nursing. Designed for the Use of Professional and Other Nurses, and especially as a Text-book for Nurses in Training. By J. C. Wilson, A. M., M. D.. Professor of the Practice of Medicine and of Clinical Medicine in the Jefferson Medical College; Visiting Physician to the Hospital of the Jefferson College and the Pennsylvania Hospital; Physician-in-Chief to the German Hospital, etc., etc. Third edition, revised and enlarged. Philadelphia: J. B. Lippincott Company. 1899.

The series of practical lessons in nursing, to which this volume belongs is too well known to need introduction or commendation. The third edition of this book on fever-nursing has been brought down to date and now includes the duties of the nurse in the care of soldiers suffering from febrile disease in camp and in transportation, as well as an article upon the oriental plague.

